# Asymptomatic Severe Acquired von Willebrand’s Syndrome in Association With a Glioblastoma Multiforme: A Case Report

**DOI:** 10.7759/cureus.11667

**Published:** 2020-11-23

**Authors:** Raphael M Laurente, Ghulam Dastagir Faisal Mohammed

**Affiliations:** 1 Neurological Surgery, Salford Royal NHS Foundation Trust, Manchester, GBR

**Keywords:** acquired von willebrand syndrome, glioblastoma multiforme, ivig

## Abstract

To our knowledge, this is the first reported case of a severe acquired von Willebrand’s Syndrome (avWS) in association with a Glioblastoma Multiforme (GBM). We report a case of a 70-year-old male who presented to the hospital with neurologic findings secondary to a thalamic mass and subsequent hydrocephalus but without any prior history of any bleeding diathesis. A biopsy and septum pellucidotomy was considered and coagulation parameters from pre-operative chemistry returned deranged. Further investigations for bleeding disorders have been performed and an avWS was diagnosed due to the low levels of factor VIII, vWF:Ag, and vWF:RiCoF. The patient responded to a single dose of IVIG and hence the contemplated procedure has been performed. Subsequently, a histopathologic diagnosis of a GBM was made and unfortunately no further treatment was pursued due to the patient's poor response to the initial surgical intervention.

## Introduction

Glioblastoma Multiforme (GBM) is a form of a primary central nervous system tumor and is the most malignant subtype of astrocytomas [1]. Its frequency is highest among elderly Caucasians especially those residing in industrialized areas [2]. It has a relatively poor prognosis and treatment is usually rendered via surgical resection with adjuvant chemoradiotherapy. Acquired von Willebrand syndrome (avWS) in relation to solid tumors is frequently being reported. However, its occurrence alongside and/or complicating a malignant glioma has not yet been extensively established. Recognition of this hematologic disorder is crucial because presentation is commonly mild in the general population. This therefore would have a great impact especially for patients in whom a surgical procedure is planned.

## Case presentation

We report a case of a hypertensive and diabetic 70-year old Caucasian male who presented with a four-week history of occipital headaches, confusion, and right-sided facial twitching. These are accompanied by a decline in cognition and difficulty with short-term memory. The patient has been taking atorvastatin, losartan, and metformin for his metabolic derangement. This patient had an unremarkable family history and no previous bleeding episodes. Prior dental appointments and hemostatic challenges in the past were as well uneventful.

**Figure 1:**
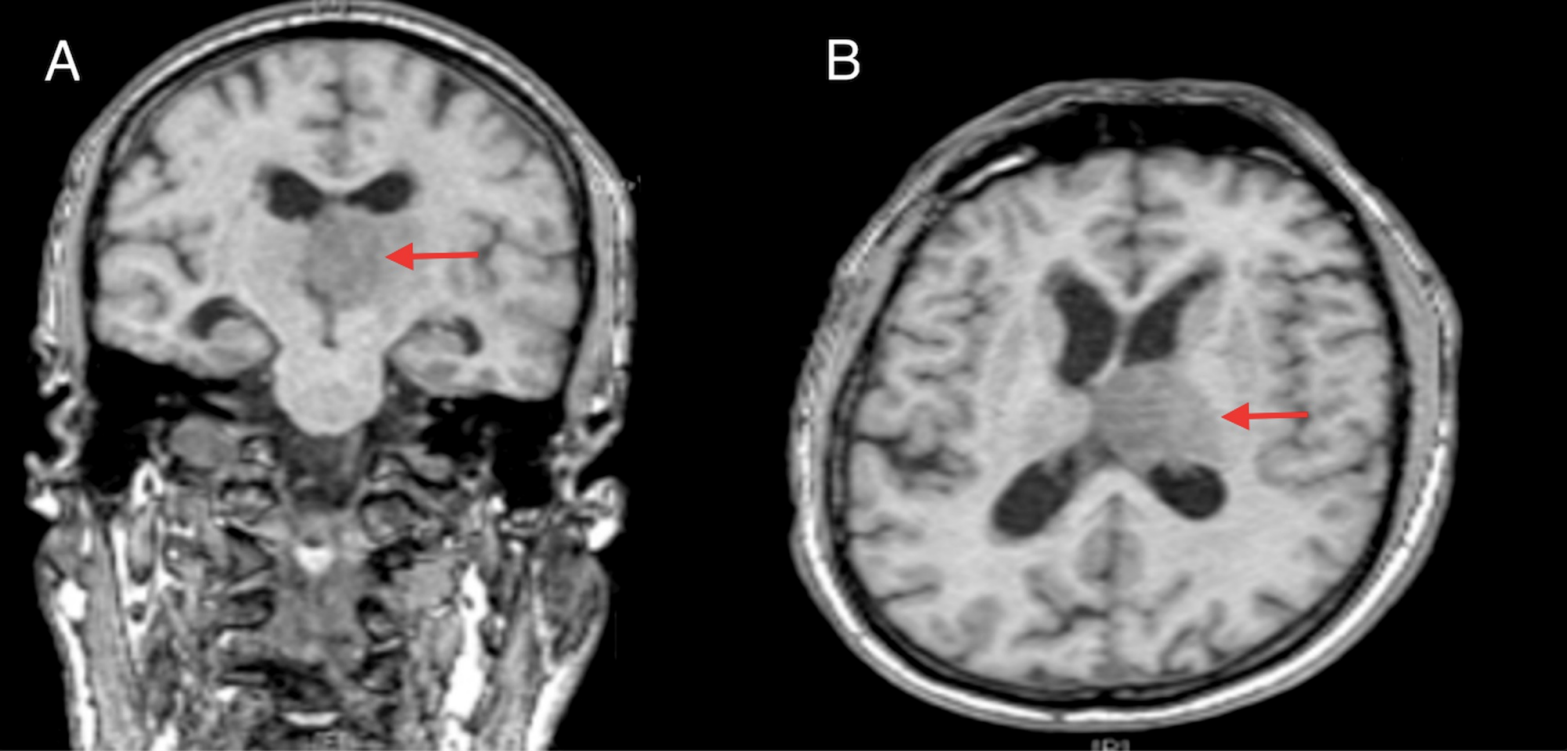
T1-weighted magnetic resonance imaging showing a left thalamic mass (red arrows): (A) coronal and (B) axial views

Examination revealed mild confusion (GCS 14, E4V4M6) but otherwise unremarkable physical and neurological findings. Review of systems were equally normal. Initial CT Brain scans have shown an iso-dense intra-axial lesion causing compression of the left foramen of Munro and subsequent hydrocephalus. Magnetic Resonance Imaging indeed confirmed a thalamic mass in which the appearance is in keeping with an infiltrative glioma (Figure 1). A biopsy with septum pellucidotomy was contemplated to ascertain the diagnosis and address the hydrocephalus.

Prior to the surgical procedure, pre-operative blood chemistry showed an isolated elevation in the activated partial thromboplastin time (aPTT) [43.5; NV:28-34]. Prothrombin time (PT) and platelet counts were within normal limits. Other hematologic investigations were therefore undertaken (Table 1).

**Table 1:** Further biochemical testing to further investigate and diagnose the hematologic disorder vWF: von Willebrand factor; RiCof: Ristocetin cofactor activity

Parameter	Normal Values	Result
Factor VIII Levels	50 – 150 U/dL	11 U/dL
vWF RiCof	50 – 200 U/dL	10 U/dL
vWF Antigen	50 – 200 U/dL	9 U/dL
Factor IX Levels	50 – 150 U/dL	101 U/dL
Factor XI Levels	50 – 150 U/dL	95 U/dL
Factor XII Levels	50 – 150 U/dL	72 U/dL
Factor VIII Inhibitor	-	Negative
Mixing Studies	-	Mixing showed correction

In the absence of a bleeding history coupled with the above results, the patient was hence diagnosed with a severe avWS. A single dose of 60g intravenous immunoglobulin (IVIg) was administered with respect to the patient’s ideal body weight. Subsequent assays have shown improvement in the vWF antigen, vWF RiCof and Factor VIII levels (Table 2). The patient underwent surgery and histopathology has subsequently confirmed a GBM (WHO IV - High Grade). Due to the persistence of this patient’s cognitive impairment and symptomatology despite the initial intervention, adjuvant therapy has been disregarded and best supportive care was considered. The patient was monitored continuously by the Hematology department upon discharge. Unfortunately, further investigations for autoimmune disorders and other myeloproliferative disorders which could have as well been associated with the aVWS were not anymore undertaken as the patient was deemed for palliation.

**Table 2:** Daily factor VIII, vWF RiCof and antigen values subsequent to a single-dose IVIG administration. Levels demonstrate a positive response from Days 1 to 5. vWF: von Willebrand factor; RiCof: Ristocetin cofactor activity

Parameter	Values prior to IVIG	Day 1 post IVIG	Day 2 post IVIG	Day 3 post IVIG	Day 4 post IVIG	Day 5 post IVIG	Day 6 post IVIG
Factor VIII Levels	11	48	109	123	256	239	338
vWF RiCof	10	<10	98	133	238	222	123
vWF Antigen	9	45	88	123	217	215	169

## Discussion

von Willebrand’s Disease (vWD) is the most common inherited bleeding disorder affecting both males and females in equal frequency, presenting mostly in childhood [3]. Occurring in an autosomal dominant fashion, the pathology is characterized by the functional, structural and quantitative alterations of the von Willebrand factor (vWF) leading to a propensity of catastrophic bleeding. vWF is most hemostatically active as a circulating high molecular weight multimeric protein ranging in size from approximately 500 to 20,000 kDaltons [4]. One of its functions is to adhere to a platelet’s (GP) Ib-IX receptor and hence facilitate platelet bridging for aggregation. This serves as the scaffolding for the formation of a hemostatic plug. In addition, vWF serves as an important carrier of Factor VIII, effectively prolonging its half-life and protecting it from enzymatic proteolysis [4]. Both of these features highlight the importance of this protein in the coagulation cascade with respect to the milieu of high shear stress.

avWS is characterized by a bleeding disorder occurring mostly in adulthood. This is usually associated with various underlying pathologies and in which there was no prior personal or family history of bleeding [5]. The presentation of an avWS is similar to a congenital vWD, although the exact pathophysiology of the former still remains to be unknown. Currently, there have been three proposed mechanisms that have been described leading to its occurrence: (1) presence of autoantibodies that form immune complexes with the large vWF multimers thereby increasing the protein’s clearance, (2) absorption of vWF onto malignant cells and hence depletion of the factor’s quantity, and (3) increased proteolysis of vWF multimers under hemorrhagic conditions brought about by cardiovascular disorders [6,7]. AvWS, however, is believed to be rare, with only 106 cases registered and treated in the United Kingdom in 2015 [8]. Worldwide prevalence studies are scant with one literature reporting a prevalence of less than 300 cases back in 2007 and most recently 446 cases in 2011 [5,9].

**Figure 2:**
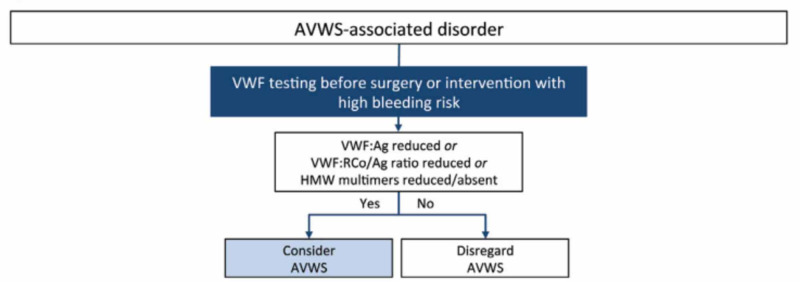
Diagnostic algorithm for an acquired von Willebrand syndrome Permission to reproduce obtained from Tiede A. Diagnosis and treatment of acquired von Willebrand syndrome. Thrombosis Research 2012 [12]. AVWS: acquired von Willebrand syndrome; VWF: von Willebrand factor; VWF:Ag: von Willebrand factor antigen; VWF:RCo/Ag: von Willebrand factor Ristocetin cofactor to antigen ratio; HMW: high molecular weight multimers

Diagnosis of an avWS relies on a high index of suspicion especially if the patient is asymptomatic. In this case, the patient’s pre-operative coagulation profile led to the diagnosis. In essence, avWS should be suspected if the von Willebrand Factor Antigen and/or the von Willebrand Ristocetin Cofactor Activity (RiCof) are low (Figure 2). The absence or the decrease in quantity of the high molecular weight multimers as observed through electrophoresis should also suggest the diagnosis [10,11]. As such, it was described that an avWS is similar to the Type 2A subtype of a congenital vWD because it decreases the protein’s activity (vWF:RiCof) and concentration alike (vWF:Ag) [6,10].

Treatment for avWS is aimed at addressing the underlying disorder as it could secondarily treat the bleeding risk [7,10,11]. For those where a curative modality is impossible or not preferred, vWF-containing concentrates, desmopressin, intravenous immunoglobulin, recombinant factor VIII, and tranexamic acid are reported to be effective agents. In this case, the patient has responded to a single dose of intravenous immunoglobulin.

There is an abundance of literature describing the association of avWS with lymphoproliferative diseases as well as myeloproliferative and cardiovascular disorders [5,7,10]. Reports which describe its relationship with solid tumors remain to be scant in literature. Nonetheless, an association with Wilm’s Tumor and adenocarcinomas have increasingly been noticed [9,10]. To our knowledge, this is the first report of an avWS in association with a Glioblastoma Multiforme.

GBM is one of the most malignant primary brain tumors catalogued as Grade 4 under the World Health Organization tumor classification. It carries a very poor prognosis albeit being common with an incidence of 2-3 per 100,000 people in Europe and North America [2]. GBMs are described as heterogenous solid tumors exhibiting necrosis, multifocal hemorrhage, and cystic and gelatinous areas [1]. Morphologically, GBMs are made up of cells characterized by extensive anaplasia, polymorphism, and anisokaryosis. GBMs are also very vascular owing to its propensity for angiogenesis.

The presentation of patients with GBM vary greatly with respect to the location of the tumor in the brain. 95% of GBMs are found in the cerebral hemispheres, and hence seizures, dysphasia, visual disturbances, apraxia, and focal neurologic deficits are often being reported [1,13]. At the present time, the standard of treatment for GBM patients is surgical resection. Due, however, to the tumor’s aggressiveness and vascularization, surgery is coupled with both chemo and radiotherapy.

Interestingly, it has been reported that vascular endothelial growth factor (VEGF), which is responsible for a GBM's increased vascularity, is positively correlated with the levels of von Willebrand Factor Antigen (vWF:Ag) [13]. Findings have shown that increased vWF:Ag levels were associated with a three-fold higher risk of death in patients diagnosed with a GBM [14]. In this instance, however, vWF:Ag levels are low and a diagnosis of an avWS is underpinned.

Unfortunately, in this case, the resolution of the avWS was not observed as the tumor was deemed unresectable and the decision has been made to give the patient the best supportive care.

## Conclusions

Diagnosis of an avWS still remains a challenge due to its late-onset of presentation and its none-to-mild symptomatology. Nonetheless, surgical patients with a chronic underlying condition presenting with derangement in coagulation parameters should prompt clinicians to investigate further and exclude avWS. Especially without a prior bleeding or family history, an avWS should always be on the top of the differentials to avoid disastrous consequences perioperatively.
